# Inhibition of sPLA_2_-IIA Prevents LPS-Induced Neuroinflammation by Suppressing ERK1/2-cPLA_2_α Pathway in Mice Cerebral Cortex

**DOI:** 10.1371/journal.pone.0077909

**Published:** 2013-10-09

**Authors:** Yanxiao Xiang, Lin Chen, Huiqing Liu, Xiaoqian Liu, Xinbing Wei, Baozhu Sun, Tian Wang, Xiumei Zhang

**Affiliations:** 1 Department of Pharmacology, Shandong University School of Medicine, Jinan, Shandong, PR China; 2 Department of Anesthesiology, Qilu Hospital, Shandong University, Jinan, PR China; 3 Department of Pharmacology, Yantai University School of Pharmacy, Yantai, Shandong, PR China; University of Medicine & Dentistry of NJ - New Jersey Medical School, United States of America

## Abstract

Neuroinflammation is involved in various central nervous system (CNS) disorders, including brain infections, ischemia, trauma, stroke, and degenerative CNS diseases. In the CNS inflammation, secretory phospholipase A_2_-IIA (sPLA_2_-IIA) acts as a mediator, resulting in the generation of the precursors of pro-inflammatory lipid mediators, such as prostaglandins (PGs) and leukotrienes (LTs). However, the role of sPLA_2_-IIA in neuroinflammation is more complicated and remains unclear yet. In the present study, we investigated the effect of sPLA_2_-IIA inhibition by specific inhibitor SC-215 on the inflammation in LPS-induced mice cerebral cortex and primary astrocytes. Our results showed that the inhibition of sPLA_2_-IIA alleviated the release of PGE_2_ by suppressing the activation of ERK1/2, cPLA_2_α, COX-2 and mPGES-1. These findings demonstrated that sPLA_2_-IIA showed the potential to regulate the neuroinflammation in vivo and in vitro, indicating that sPLA_2_-IIA might be a novel target for the treatment of acute neuroinflammation.

## Introduction

Neuroinflammation is a process principally associated with an activation of astrocytes and microglia by inflammatory mediators in central nervous system (CNS) disorders, including brain infections, brain inflammation, ischemia, trauma, stroke, and degenerative CNS diseases, such as Alzheimer disease (AD), Parkinson’s disease (PD), and axonal degeneration in multiple sclerosis (MS) [1]. Cytokines, chemokines, reactive nitrogen species [2], [3] and prostaglandins (PGs) [4] are upregulated in the process of inflammation. Prostaglandin E_2_ (PGE_2_) is one of the central inflammatory markers and key mediators of neuroinflammation induced by ischemia and bacterial infection [5], [6].

In the CNS inflammation, secretory phospholipase A_2_-IIA (sPLA_2_-IIA) is known as a mediator to hydrolyze fatty acids from the *sn*-2 position of membrane phospholipids, resulting in the generation of free arachidonic acid (AA) and lysophospholipids which are the precursors of pro-inflammatory lipid mediators, such as prostaglandins (PGs) and leukotrienes (LTs) [7]. As the most abundant isoform of sPLA_2_ [8], [9], sPLA_2_-IIA is originally isolated from inflammatory fluids and cells and identified to play a prominent role in inflammation and infectious diseases [10] [11]. sPLA_2_-IIA is generally localized in the hippocampus and cerebral cortex in brains of ischemia and AD [12], [13]. Due to its phospholipase enzymatic activity, sPLA_2_-IIA interacts with cytosolic phospholipase A_2_ (cPLA_2_) to supply AA to downstream cyclooxygenase-2 (COX-2) for PGE_2_ biosynthesis [14]. However, the exact role of sPLA_2_-IIA in neuroinflammation remains unclear.

Mitogen-activated protein kinases (MAPK), including ERK1/2 and p38, are critical regulators of proinlammatory cytokines (PGE_2_, TNF-α, IL-6 and IL-1ß) during inflammation. The biosynthesis of PGE_2_ is regulated by cPLA_2_α [14], which is activated by MAPK. Recent work has demonstrated that activation of p38 kinase by thrombin regulated cPLA_2_ activation and lipid release in human platelet [15], ATP or ionomycin-stimulated phosphorylation of ERK1/2 is required for the activation of cPLA_2_ in Madin-Darby canine kidney (MDCK) cells [16]. However, it is not known whether ERK1/2 or p38 kinase plays a role in the deleterious effect of LPS on CNS. In the CNS, activated astrocytes and microglia are the main sources of PGs during neuroinflammation [17], [18]. Astrocytes are the predominant immunocompetent cells in the brain where they act not only as central effectors of cerebral immune and inflammatory diseases such as Parkinson's disease [19] and stroke [20], but also as an important sensor of invading pathogens including bacteria and viruses [21]. Bacterial endotoxins can induce the synthesis and release of proinflammatory mediators, i.e., chemokines and eicosanoids in astoryctes [22]. Previous study has shown that astrocytes are not only a source of PGE_2_, but also a target of its modulatory action in immune and inflammatory processes in CNS [23].

In the present study, RT-PCR analysis revealed that LPS exposure led to a time- and dose- dependent increase of sPLA_2_-IIA mRNA expression in murine primary astrocytes but not in primary microglial cells. Therefore, we used primary astrocytes to study the role of sPLA_2_-IIA in lipopolysaccharide (LPS)-induced acute neuroinflammation in vitro. Furthermore, the neuroinflammation in vivo induced by the intracerebroventricle microinjection (i.c.v.) of LPS was employed to explore the regulation of MAPK-cPLA_2_α–PGE_2_ pathways by sPLA_2_-IIA in mice cerebral cortex.

## Materials and Methods

### Materials

The following chemicals and materials were purchased, LPS from *Escherichia coli* 0111:B4 (Sigma, St. Louis, USA), the specific group IIA sPLA_2_ inhibitor, SC-215 (Santa Cruz, California, USA), cPLA_2_α inhibitor, AACOCF3 (Calbiochem, SanDiego, CA), antibody against sPLA_2_-IIA (BioVendor, Candler, NC), antibody against phospho-ERK1/2, phospho-p38 and cPLA_2_α, and the total protein, COX-2, mPGES-1 (Cell signaling Technology, Danvers, MA), anti-glial fibrillary acidic protein (GFAP) monoclonal antibody for astrocytes (Bioss, Beijing), the p38 MAP kinase inhibitor(SB203580), the MEK_1_/MEK_2_ inhibitor (U0126) (Sigma Chemical Co. St. Louis, MO), Dulbecco's modified Eagle's medium (DMEM) and other materials for cell culture (Gibco, Carlsbad, USA).

### Mice and production of CNS inflammation

Zero to one-day-old Wistar rat pups were purchased from the Experimental Animals Center, Shandong University. Male C57BL/6 mice (6–8 weeks old) were purchased from Beijing Vital River Company. Mice were housed in polypropylene cages and maintained at 21°C under a reverse-phase 12 h light,12 h dark cycle with free access to water and rodent chow. All the protocols of experiments were approved by the Institutional Care and Use Committee of Shandong University and performed according to the Guide for the Care and Use of Laboratory Animals published by the US National Institutes of Health.

Intracerebroventricle microinjection was performed as previously described [24] with minor modifications. LPS was dissolved in sterile normal saline at a concentration of 1 mg/ml. On test day, mice were anesthetized by intraperitoneal injection (i.p.) with mixture of ketamine (100 mg/kg) and xylazine (10 mg/kg) and placed in a stereotaxic apparatus. The scalp was shaved and sterilized and a burr hole was drilled according to the following coordinates: 2.0 mm lateral to the midline and 1 mm caudal to bregma. Mice were received i.c.v. injection with 2.5 µg LPS [25] or subjected to i.c.v. injection with 1.218 µg SC-215901 1 hour before the LPS injection using a Harvard Apparatus syringe pump Mice in vehicle control group received i.c.v. injection with 2.5 µL saline or 2 µL DMSO. The concentration of LPS and SC-215 was identified to induce optimal responses in preliminary experiments and has been used by a number of other laboratories [26].The solutions were injected into the ventricle over a 2-min period, and the needle was left in the injection site for 4 minutes before it was slowly removed. The wound was sutured and the mice were euthanized at various time (10, 20, 30, 60 minutes) thereafter and the brains were removed, dissected and stored at −80°C.

### Primary astrocyte cultures

Primary astrocyte cultures were prepared from the cortices of 0 to 1-day-old Wistar rat pups as previously described [27] with minor modifications. Briefly, cerebral cortices were dissected and meninges were removed in Petri dishes with D-Hanks balanced salt solution. The tissues were thoroughly diced with scalpels and suspended in 10 volumes 0.25% (w/v) trypsin and incubated for 20 min at 37°C. The same volume contains 10% (v/v) fetal bovine serum in high glucose DMEM was added to stop cleavage. After centrifugation (5 min, 200 g, 25°C), the supernatant was discarded and the pellet was dispersed in new medium containing high glucose DMEM (added 5.5 mM glucose) with addition of 20% FBS, 100 units/mL penicillin, and 100 µg/mL streptomycin. Then, the cell suspension was filter through 85 µm nylon mesh and transferred to poly-L-lysine coated 75 cm^2^ culture flasks. The culture medium was changed after 24 h and then every 3 days afterwards. When the cells became confluent after 2 weeks of culturing, flasks were shaken at 200 rpm on an orbital shaker at 37°C for 3 h to remove non-astrocyte cells. Cells were >95% positive for GFAP, the astrocytic marker. Cells were washed trypsinized and subcultured in 6-well (1.0×10^6^) plates, selected cells were pretreated with varying concentrations of SC-215, U0126 or SB203580 for 2 hours. DMSO, at concentrations similar to comparative experiments, was used as an appropriate control and never exceeded 0.1%. Selected cells were then treated with LPS and the incubations ranged from 5 minutes to 240 minutes, as indicated in the text.

### Reverse transcription-polymerase chain reaction (RT-PCR) analysis

After incubation, media was removed and cultured cells were washed with phosphate-buffered saline (PBS) twice. Total RNA was prepared from cells by using the Trizol® reagent (Invitrogen Corporation, Carlsbad, CA, USA) according to the manufacturer's protocol. cDNA was prepared using reverse transcriptase originating from the Superscript™-III kit (Invitrogen) with 2.5 µg total RNA and oligo dT. The sequences of PCR primers were as follows: sPLA2-IIA, upper primer 5'-AAGGAAGCCGCACTCAGTTA-3' and lower primer 5'-GGCAGCAGCCTTATCACACT-3'; GAPDH, upper primer 5'-CAAGGTCATCCACGACCACT-3' and lower primer 5'-CCAGTGAGTTTCCCGTTCAG-3'. GAPDH expression was used as an internal calibrator for equal RNA loading and to normalize relative expression data for sPLA_2_-IIA gene analyzed.

### Western blot analysis

Protein concentration was measured by using a BCA protein assay kit and loaded on the 10% Acrylamide-SDS-PAGE at 120 V in duplicates for electrophoresis and then transferred to NC (Nitrocellulose) membranes at 200 mA for one hour. Membranes were then blocked in Tris-buffered saline, pH 7.4 (TBS) with 0.1% Tween 20 (TBS-T) containing 5% non-fat milk for one hour at room temperature and then incubated with primary antibodies against TLR4/CD284 (1∶1000), sPLA_2_-IIA (1∶500); phospho-ERK1/2 and ERK1/2 (1∶10000), phospho-cPLA2α and cPLA2α (1∶1000) and GAPDH (1∶1000) overnight at 4°C. After washing with TBS-T, blots were incubated with secondary antibodies for one hour at room temperature. Immunolabeling was detected by ECL (Millipore). For quantification, blots were scanned and the band intensities were measured as optical density using the Quantity One program (BioRad, Hercules, CA) and normalized against GAPDH loading control.

### Determination of PGE_2_ concentration

Samples from the cortex were collected at different time as described above. The samples were weighted and homogenized. Then the PGE_2_ concentration of the supernatant was measured by means of ELISA-based assay (Cayman Chemical) [28].

### Statistical Analysis

All data are presented as means ± S.E.M. from three or more independent experiments. For time-response and SC-215 dose-response studies, statistical analysis was assessed by either Student’s t test or one-way ANOVA followed by post hoc Bonferroni tests. For other expreriments, statistical analysis was assessed by two-way ANOVA followed by post hoc Bonferroni tests to compare differences between the various treatment groups. In all cases, differences with *p* value < 0.05 were regarded as statistically significant.

## Results

### Inhibition of sPLA_2_-IIA reduces PGE_2_ production in LPS-stimulated neuroinflammation

**Figure 1:**
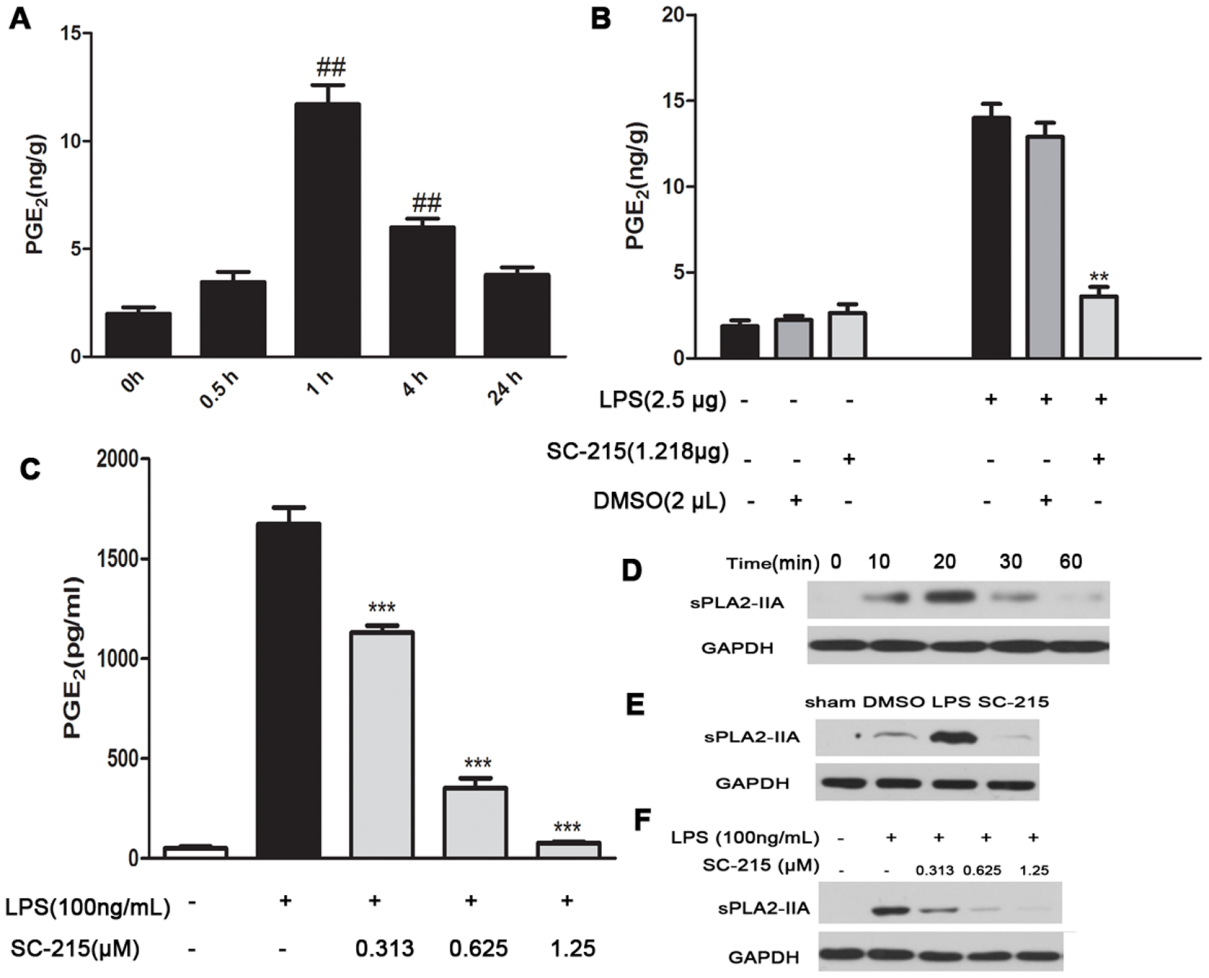
sPLA_2_-IIA inhibition prevents the release of PGE_2_ in LPS-stimulated brain and astrocytes. (A) Mice (n = 4) were given i.c.v. injection with LPS (2.5 µg) for 0, 0.5, 1, 4, 24 hours and PGE_2_ in the cortex were detected by ELISA. Since the levels of PGE_2_ in the cortex with LPS (2.5 µg) for 1 hour were significantly higher than the control (0 hour), we chose the stimulation of LPS (2.5 µg) for 1 hour in the following experiments in vivo. (B) Inhibition of sPLA_2_-IIA by SC-215 remarkably reduced the production of PGE_2_ in the cortex treated with LPS (2.5 µg) for 1 hour. (C) SC-215 lowered LPS-elevated PGE_2_ levels in a dose dependent manner in primary astrocytes. (D) The sPLA_2_-IIA protein expression was detected by Western Blot in mice cerebral cortex after treatment with LPS (2.5 µg) for 0, 10, 20, 30, 60 minutes. (E) The LPS-induced sPLA_2_-IIA upregulation was significantly suppressed by the SC-215 in cerebral cortex. (F) The LPS-induced sPLA_2_-IIA protein expression was significantly reduced by SC-215 in a dose-dependent manner in primary astrocytes. Values represent the mean ± S.E.M. of results from five animals in each group. One symbol, p<0.05; two symbols, p<0.01; three symbols, p<0.001. Symbols indicate comparison versus either (^#^) control or (*) LPS.

In order to understand the anti-inflammatory effect of sPLA_2_-IIA inhibition, we first quantified the PGE_2_ metabolites in mice cortex. I.c.v. administration of LPS induced PGE_2_ generation (Fig. 1A) and a concomitant release of sPLA2-IIA in the cortex of mice (Fig. 1D). To investigate the effect of sPLA2-IIA on PGE_2_ release, a highly specific sPLA_2_-IIA inhibitor, SC-215, was presented to the lateral ventricle together with LPS. SC-215 is reported to block the enzymatic activity of sPLA_2_-IIA via highly selective binding to the active center of sPLA_2_-IIA, with an IC_50_ as low as 29 nM in rats [29], [30]. Our results showed that the addition of SC-215 (1.218 µg) with LPS (2.5 µg) showed a nearly 90% decrease in sPLA_2_-IIA expression (Fig. 1E). As expected, PGE_2_ production were significantly inhibited (>75%) in the cortex of SC-215-treated mice at this time point (Fig. 1B). These results indicated that sPLA_2_-IIA was involved in LPS-stimulated acute neuroinflammation via mediating PGE_2_ generation. Attenuated the sPLA_2_-IIA expression and generation of PGE_2_ were confirmed in vitro in primary rat astrocytes that were pretreated with SC-215 then exposed to LPS for 4 hours (Fig. 1, C and F). Since our preliminary results indicated that LPS exposure led to a time- and dose-dependent increase of sPLA_2_-IIA mRNA expression in murine primary astrocytes (data not shown) but not induce increase of sPLA_2_-IIA mRNA or protein expression in the primary microglia (Fig. S1), we used astrocytes to simulate similar inflammatory situation in vitro to study the role of sPLA_2_-IIA in LPS-stimulated route of PGE_2_ biosynthetic pathway. Western blotting (Fig. 1F) showed that SC-215 (1.25 µM) reduced the sPLA_2_-IIA protein expression by 85–90%. SC-215 treatment lowered the elevated PGE_2_ level induced by LPS in a dose dependent manner in astrocytes (Fig. 1C).

### sPLA_2_-IIA inhibition suppresses the activation of ERK1/2 and cPLA_2_α induced by LPS

**Figure 2:**
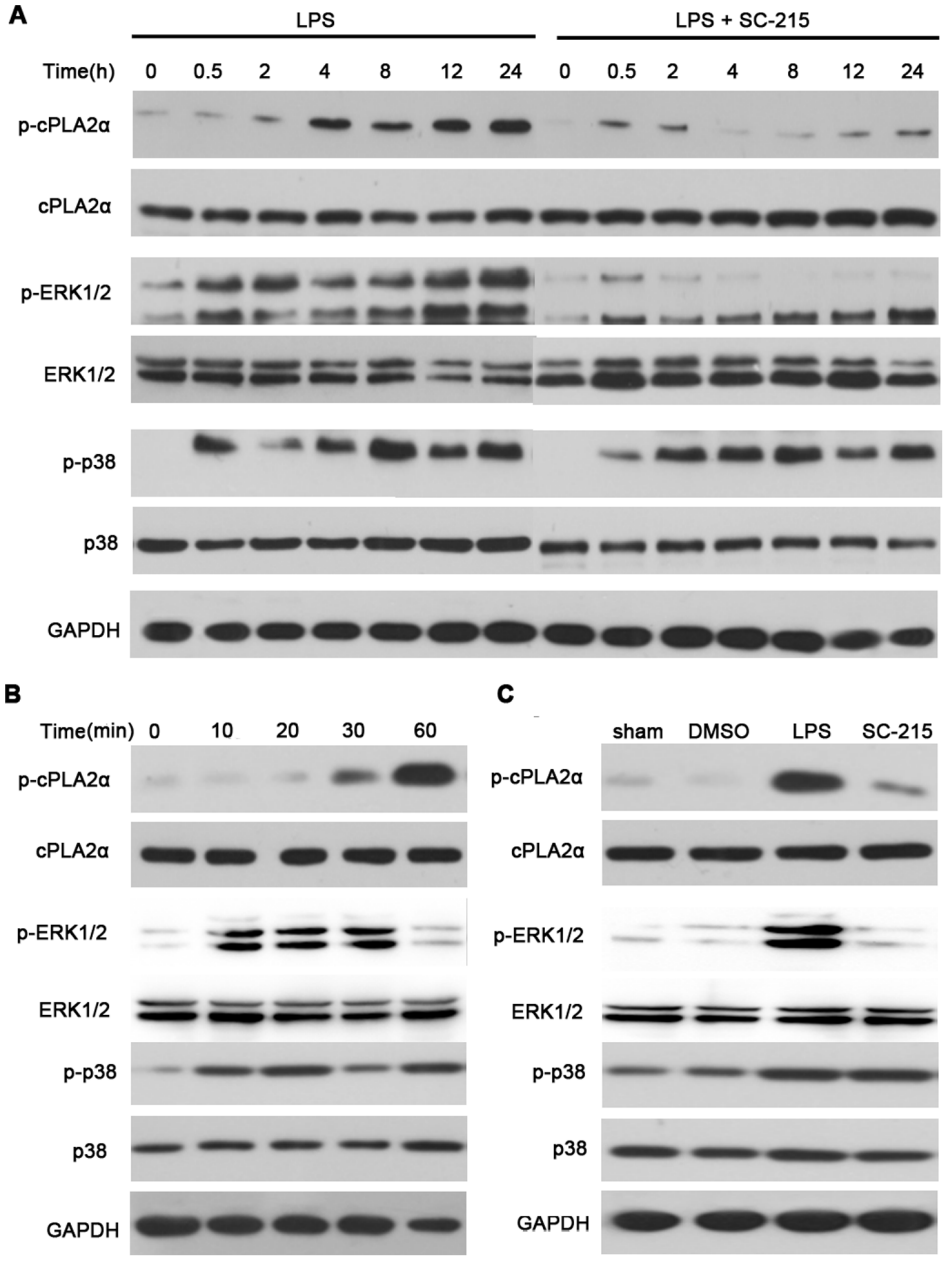
Inhibition of sPLA_2_-IIA by SC-215 reduces LPS-induced phosphorylation of ERK1/2 and cPLA_2_α. (A) Astrocytes (n = 3) were incubated for 0 to 4 hours with 100 ng/mL LPS or pretreated with 1.25μM SC-215 for 2 hours before LPS stimulation. (B) Mice (n = 4) were given i.c.v. injection with LPS (2.5 µg) alone for up to 1 hour or injected with SC-215 (1.218 µg) 1 hour before LPS stimulation. (C) In the subsequent experiments, the cortex was obtained 10 mintues or 1 hour later to study the effect of SC-215 on LPS-induced phosphorylated ERK1/2, p38 or cPLA_2_α respectively. Expression of phosphorylated (p) and total cPLA_2_α, ERK1/2, and p38 was assessed by Western blotting as described in methods.I.

It is well established that cPLA_2_α is essential for PGE_2_ production by supplying arachidonic acid for eicosanoid biosynthesis [31]. As reported previously, MAP kinase contributed to phosphorylation of cPLA_2_α in response to several stimuli [32], [33]. To elucidate the mechanisms through which sPLA_2_-IIA regulates LPS-induced PGE_2_ generation, we examined the effect of deleting sPLA_2_-IIA on phosphorylation of cPLA2α and its upstream MAP kinases, ERK1/2 and p38 in vitro and in vivo. Rat primary astrocytes were pretreated with or without SC-215 (1.25 µM) for 2 hours and then incubated with 100 ng/mL LPS for up to 4 hours (Fig. 2A), mice were given i.c.v. injection with LPS (2.5 µg) for up to 1 hour (Fig. 2B), phosphoprotein expression was examined by Western blotting. The effect of 1.218 µg SC-215 on cPLA_2_α activation at 1 hour and MAP kinases activation at 10 minutes after LPS stimulation in mice cortex were also measured (Fig. 2C). The data showed that LPS-stimulated cPLA_2_α phosphorylation in a time-dependent manner with a peak within 30 minutes and sustained to reach a maximum at 4 hours in astrocytes. In contrast, in SC-215-pretreated astrocytes phosphorylation was also detectable within 5–10 minutes, but the further rise in phosphorylation and sustained phosphorylation after 10 minutes were markedly attenuated. Phosphorylation of ERK1/2 proceeded that of cPLA_2_α and was apparent within 5 minutes of LPS stimulation in both control or SC-215 pretreated astrocytes. This phosphorylation was sustained to 4 hours in LPS-stimulated astrocytes. In contrast, ERK1/2 phosphorylation declined rapidly in SC-215-pretreated astrocytes and was barely detectable by 10 minutes (Fig. 2A). There was no phosphorylation of cPLA2α or ERK1/2 up to 4 hours in the absence of LPS (data not shown). Phosphorylation of p38 MAP kinase was detectable in the absence of SC-215 at all the indicated time points and increased in a similar manner in LPS-treated and SC-215-pretreated astrocytes from 2 hours after LPS stimulation (Fig. 2A). Our in vivo study found that LPS caused a time-dependent induction of phospho-cPLA_2_α, phosphorylation of ERK1/2 preceded that of cPLA_2_α and increased to a maximum at 10 minutes in LPS treated brain tissue. The residual phosphorylation was still detectable 30 minutes after stimulation and returned to baseline by 60 minutes (Fig. 2B). In subsequent experiments, changes in the activation of these downstream molecules in cortex after SC-215 treatment were examined. The phosphorylation of cPLA_2_α at 1 hour and phosphorylation of ERK1/2 at 10 minutes were significantly prevented in mice treated with SC-215 prior to LPS challenge, but the induction of phospho-p38 at 10 minutes were not affected upon SC-215 pretreated and remained similar to LPS challenge values (Fig. 2C).

### Inhibition of sPLA_2_-IIA induced protection by regulating ERK1/2 dependent cPLA_2_α activation

**Figure 3:**
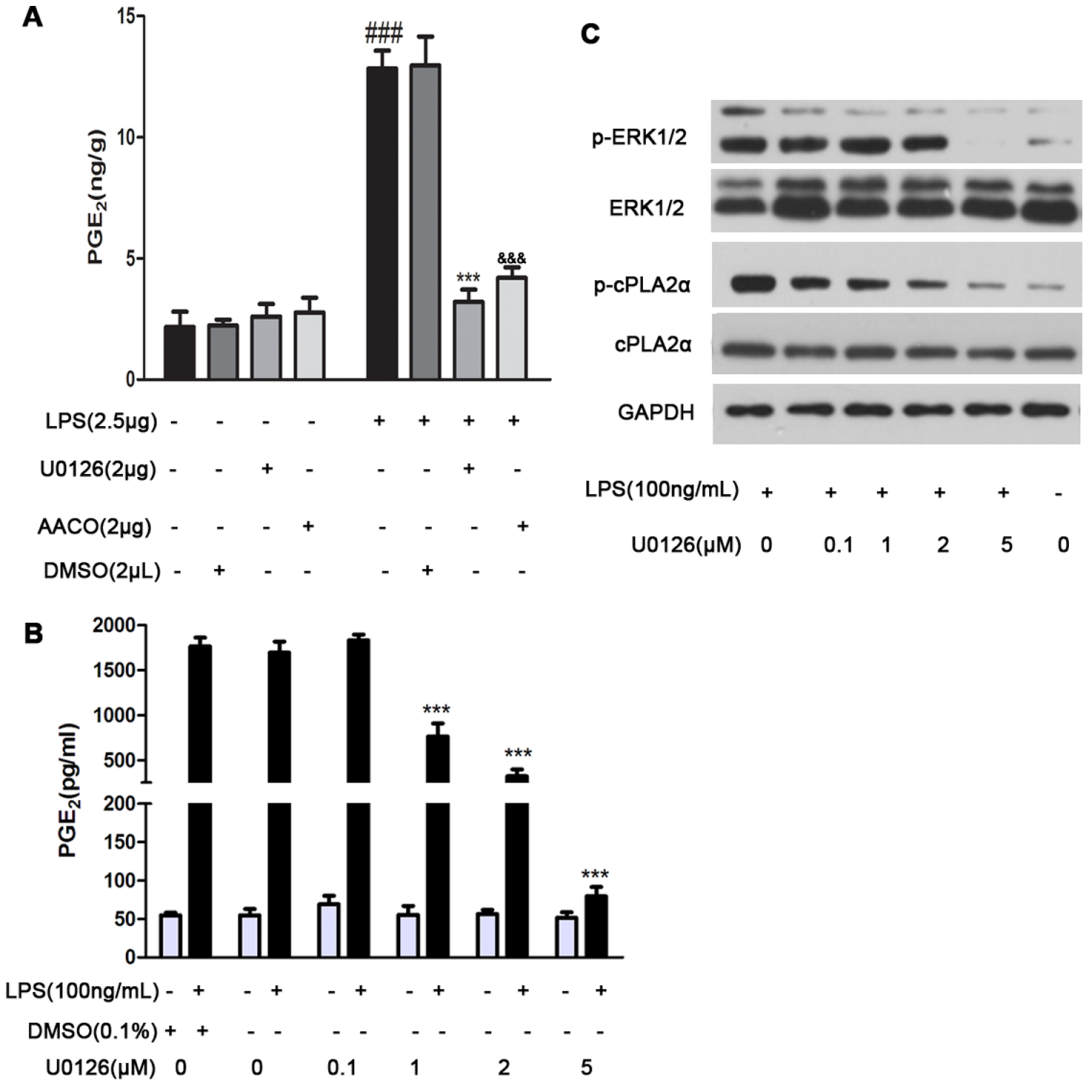
ERK1/2 regulates the activation of cPLA_2_α and the generation of PGE_2_ in LPS-induced mice and astrocytes. (A) Mice (n = 4) were given i.c.v. injection with LPS (2.5 µg) alone or injected with AACOCF3 (2 µg) or U0126 (2 µg) 1 hour before LPS stimulation for 1 hour. (B) The in vitro effects of U0126 on PGE_2_ production and (C) phosphorylation of ERK1/2 and cPLA_2_α in primary rat astrocytes were determined. Astrocytes (n = 3) were treated with U0126 (01, 1, 2 and 5 µM) for 2 hours before LPS (100 ng/mL) stimulation for up to 24 hours. The PGE_2_ levels in tissue homogenate at 1 hour after LPS stimulation (A) or culture supernatants at 24 hours after LPS stimulation (B) were determined by using ELISA-based assay as described in methods, the phosphorylation of ERK1/2 and cPLA_2_α in the cell lysates at 4 hours after LPS challenge (C) were determined by western blot as described in methods. Values represent the mean ± S.E.M. of results from five animals in each group. *One symbol, p<0.05; two symbols, p<0.01; three symbols, p<0.001.* (^#^), significant comparisons for LPS versus control ; (*), significant comparisons for U0126 versus LPS; (^&^), significant comparisons for AACOCF3 versus LPS.

Since our results indicated that sPLA_2_-IIA amplified the action of cPLA_2_α and ERK1/2, we then evaluated the role of MAP kinase in cPLA_2_α activation and PGE_2_ generation elicited in response to sPLA_2_-IIA signaling. Mice were i.c.v. injection of an inhibitor of cPLA_2_α activity (AACOCF3) or MEK1/MEK2 inhibitor (U0126) and then exposed to LPS for 1 hour. Astrocytes were pretreated with p38 MAP kinase inhibitor (SB203580) or U0126 before LPS to determine if MAP Kinase altered astrocytes phosphorylation of cPLA_2_α and release of PGE_2_ following LPS stimuli. As shown in Fig 3A, AACOCF3 and U0126 infusion did decrease the brain concentrations of PGE_2_ by 3.5- and 4-fold, respectively. In agreement,the in vitro study showed that U0126 dose-dependently attenuated LPS-induced PGE_2_ release at 24 hours from astrocytes. The inhibition was apparent at doses greater than 1 µM and was complete with 5 µM (Fig. 3B). In contrast, SB203580 did not attenuate LPS-induced PGE_2_ release (data not shown ). The LPS-induced increase in ERK1/2 and cPLA_2_α phosphorylation at 4 hours was ablated in astrocytes by U0126 in a dose-dependent fashion (Fig.3C), but pretreated with SB203580 did not affect PGE_2_ production induced by LPS ( data not shown ). These results imply that activation of ERK1/2 but not p38 kinase by LPS regulates cPLA_2_α activation and PGE_2_ release in astrocytes and mice cerebral cortex.

### Inhibition of sPLA_2_-IIA prevents LPS-induced activation of COX-2 and microsomal PGE synthase-1 (mPGES-1)

**Figure 4:**
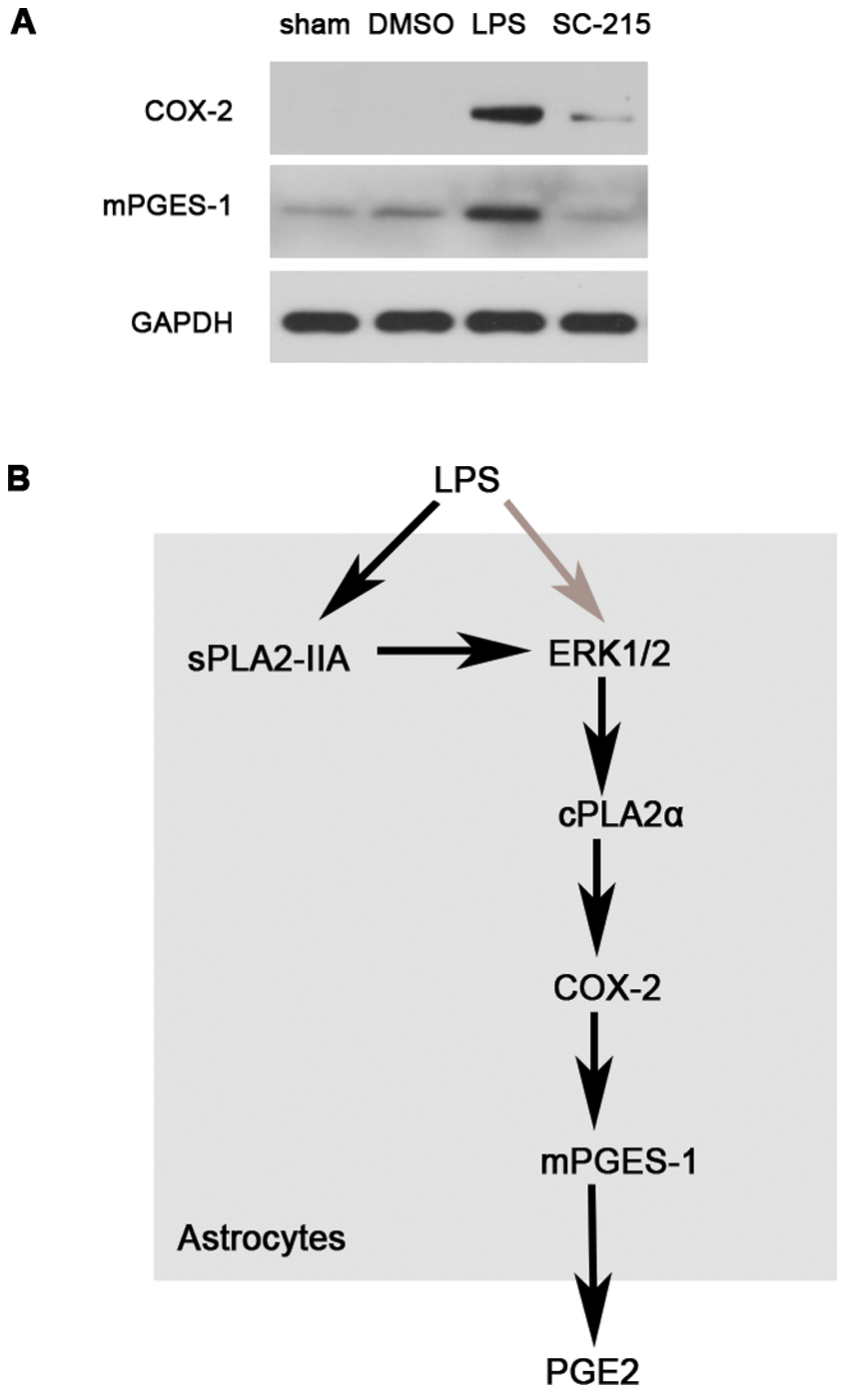
sPLA_2_-IIA inhibition prevents LPS-induced expression of COX-2 and mPGES-1. (A) Mice (n = 4) were given i.c.v. injection with LPS (2.5 µg) alone or injected with SC-215 (2.5 µg)1 hour before LPS stimulation for 1 hour. The cortex was obtained 1 hour later to study the effect of SC-215 on LPS-induced COX-2 and mPGES-1 expression respectively. Protein exptessions were assessed by Western blotting as described in methods.(B) Possible molecular mechanism involved in LPS-induced PGE_2_ production in astrocytes. LPS activates the sPLA_2_-IIA, leading to increased levels of phospho-ERK1/2. The accumulation of phosphor-ERK1/2 levels modulates the downstream activates of cPLA_2_α, cPLA_2_α regulates COX-2 and mPGES-1 enzyme activations which lead to the production of PGE_2_. Black solid arrows represent the novelties of the present study. LPS, lipopolysaccharide;, secretory phospholipase A_2_-IIA; cPLA_2_α, cytosolic phospholipase A_2_α; COX-2, cyclooxygenase-2; PGE_2_, prostaglandin E_2_; mPGES-1, microsomal PGE synthase-1.

Since synthesis of PGE_2_ is catalyzed by enzymes such as cyclooxygenases (COX) and microsomal PGE synthase-1 (mPGES-1), we next investigated the effect of sPLA_2_-IIA inhibition on LPS-induced expression of these proteins in mice cortex. The constitutive COX-1 enzyme was expressed only at low levels in cortex and was not significantly altered by LPS treatment (data not shown). In contrast, the COX-2 enzyme was not expressed under basal conditions in mice cortex but was induced by LPS stimulation and was downregulated in the presence of SC-215 (Fig. 4). The mPGES-1 is the final enzyme in the cascade to ultimately generate PGE_2_. In mice cortex, mPGES-1 was found to be constitutively expressed and was upregulated upon LPS treatment, the change was significantly prevented by sPLA_2_-IIA inhibition (Fig. 4).

## Discussion

Our results revealed that the inhibition of sPLA_2_-IIA by SC-215 showed an ability to diminish the inflammatory response after stimulation with LPS. LPS, a Gram-negative bacterial cell wall component with high pro-inflammatory capabilities, is known to induce various cytotoxic and inflammatory changes via activation of PLA_2_ and AA metabolism. Several studies have shown that the exposure of different organs, such as liver [34] and heart [35], to bacterial endotoxin LPS leads to activation of PLA_2_, which releases AA from membrane lipids subsequent to LPS stimulation. Further, COX and LOX enzymes metabolize AA into PGE_2_, TXB and LTB4, major inflammatory lipid mediators. As the pre-eminent AA metabolite, PGE_2_ is implicated in various inflammatory diseases such as sepsis [36], allodynia [37] and fever [38]. Therefore, regulation of PGE_2_ and other AA metabolites by inhibiting or decreasing the expression of AA metablic enzymes such as PLA_2_, COX-2 could be an effective mechanism for the prevention of inflammation and related pathologies. While there is a large body of data on the mechanisms of AA under the CNS disease conditions, there is limited information on the mechanisms by which the LPS evokes the production of PGE_2_ in acute neuroinflammation. The present study was undertaken to provide the first insight into the LPS induced PGE_2_ release in mice cerebral cortex and into the molecular mechanism by which sPLA_2_-IIA elicits the PGE_2_ generation.

sPLA2-IIA is widely expressed in mammalian cells, snake and bee venom. It has been shown to be implicated in the CNS pathologies including Alzheimer’s disease, Parkinson's disease, multiple sclerosis, and stroke. Although there is substantial interest in the role of sPLA_2_-IIA in the neuroinflammatory responses in CNS, limited report has described the production of sPLA_2_-IIA in cortex during LPS challenge. In the present study, we demonstrated for the first time that i.c.v. injection of LPS induced sPLA_2_-IIA protein expression from mice cerebral cortex in a time-dependent manner, SC-215 completely suppressed LPS induced sPLA_2_-IIA expression, inhibition of sPLA_2_-IIA was found to cause a significant decrease of PGE_2_ generation. Up-regulation of sPLA_2_-IIA protein and immunoreactivity is inducible following various stimuli in a wide variety of inflammatory cells such as macrophages [39], mast cells [40], human eosinophils [41]. The expression pattern of sPLA_2_-IIA in the CNS is complex, thus, several possible mechanisms could explain the in vivo neuroprotective effect of ablating sPLA_2_-IIA in the i.c.v. LPS model. In CNS, sPLA_2_-IIA is present mainly in astrocytes. In line with this result, our RT-PCR analysis revealed that LPS exposure led to a time- and dose-dependent increase of sPLA2-IIA mRNA expression in murine primary astrocytes (data not shown). Thus, we directly tested the hypothesis that sPLA_2_-IIA was critical to astrocytes activation and release of PGE_2_. We observed that LPS evoked marked PGE_2_ generation in primary astrocytes in a time-dependent fashion accompanied by release of sPLA_2_-IIA (data not shown). We reasoned that pharmacologic suppression of sPLA_2_-IIA in cultures should produce the same results as in vivo. Indeed, SC-215 dose-dependently suppressed PGE_2_ generation in astrocytes (Fig. 1C). Given the concordance between the in vitro and in vivo findings, these data strongly implicate that the inhibition of sPLA_2_-IIA alleviates the release of PGE_2_ following the neuroinflammation induced by LPS.

To our knowledge, cPLA_2_α is the only known PLA_2_ with pronounced specificity for *sn-2* arachidonoyl-containing phospholipids [42], [43] and is generally thought to play a crucial role in maintaining cellular AA levels [44]. Recently study reveals that the phosphorylation of cPLA_2_α Ser505 by MAP kinases is essential for agonist-induced AA release [45]. Using inhibitors of p38 MAP kinase and MEK1/MEK2, we demonstrated that ERK1/2, not p38 was required for the sequential activation of cPLA_2_α and further release of PGE_2_ induced by LPS. U0126 could suppress LPS-stimulated activation of cPLA_2_α and block PGE_2_ release from astrocytes in a dose-dependent manner. Moreover, U0126 could also attenuate LPS-evoked PGE_2_ release in mice cerebral cortex (Fig. 3). While the early phases of ERK1/2 and cPLA_2_α were intact in SC-215 pretreated astrocytes and their sequential sustained phosphorylation was attenuated (Fig. 2). The results was compatible with experiments in vivo, strongly confirming that sPLA_2_-IIA regulates the activation of cPLA_2_α by sustaining the activation of ERK1/2, which is critical for the sustained phosphorylation of cPLA_2_α in response to LPS stimulation.

COX-2 is a crucial enzyme for the production of PGE_2_ during inflammation and it has been shown to be dependent upon the activation of cPLA_2_α via a feedback mechanism as cPLA_2_α provides the substrate (AA) for COX-2 [46]. AA is further metabolized by COX-2 into prostaglandin G_2_ (PGG_2_), which upon conversion to PGH_2_ serves as the substrate for specific prostaglandin synthases such as mPGES-1 [22]. Thus, inhibition of COX-2 in combination with down-stream mPGES-1 appears to be responsible for the decreased biosynthesis of PGE_2_ and the alleviation of inflammation. In the present study, we investigated whether the expression of COX-2 and mPGES-1 is regulated by sPLA_2_-IIA. Our results demonstrate that SC-215 significantly decreased the expression of these two enzymes (Fig. 4), confirming that the inhibition of sPLA_2_-IIA suppressed the synthesis of PGE_2_ and the inflammation induced by LPS in mice.

In conclusion, our data provides the first dissection about the role of sPLA_2_-IIA in the neuroinflammation induced by LPS in C57BL/6 mice cerebral cortex and primary astrocytes. We demonstrate a role of sPLA_2_-IIA in amplifying CNS inflammation, especially in regulating the activation of cPLA_2_α by sustaining the activation of ERK1/2. The elevated signaling of the sPLA_2_-IIA-ERK1/2-cPLA_2_α-COX-2-mPGES-1 pathway contributes to PGE_2_ overexpression and secretion in primary astrocytes and mice cortex. This amplification can be reversed by sPLA_2_-IIA specific inhibition,which indicated that sPLA_2_-IIA mediated signaling pathway might be a potential target for the treatment of the acute neuroinflammation.

## Supporting Information

Figure S1 **Primary microglia is not capable of responding to LPS in the production of sPLA_2_-IIA at the transcriptional and translational levels.** (A) and (C) show the mRNA expression of sPLA_2_-IIA and (B) and (D) show the corresponding protein data. (A and B) Primary microglia (n = 3) were stimulated with LPS (1μg/mL) for 0, 0.2, 0.5, 2, 4, 24, 48 hours. (C and D) Primary microglia (n = 3) were treated with LPS (0, 1, 10, 100, 1000ng/mL) for 24 hours. The mRNA and protein levels of sPLA_2_-IIA were determined by western blot as described in methods.
(TIF)
