# Supplementary figures and images for: Inhibition of sPLA2-IIA Prevents LPS-Induced Neuroinflammation by Suppressing ERK1/2-cPLA2α Pathway in Mice Cerebral Cortex
Source: PLoS One. 2013 Oct 9;8(10):e77909. doi: 10.1371/journal.pone.0077909 (PMC3793966; doi:10.1371/journal.pone.0077909)

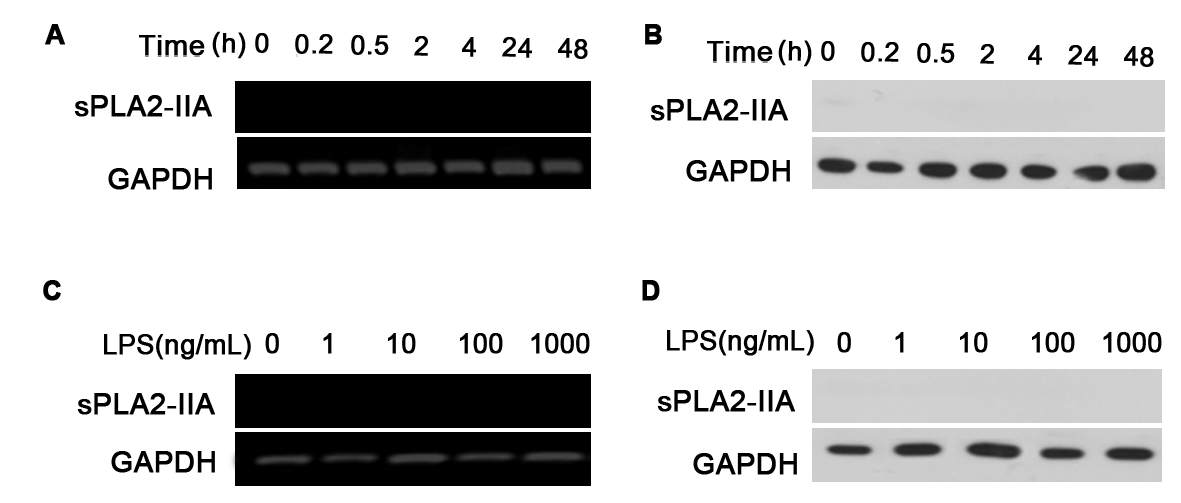

Supplement: Figure S1 — Primary microglia is not capable of responding to LPS in the production of sPLA2-IIA at the transcriptional and translational levels. (A) and (C) show the mRNA expression of sPLA2-IIA and (B) and (D) show the corresponding protein data. (A and B) Primary microglia (n = 3) were stimulated with LPS (1μg/mL) for 0, 0.2, 0.5, 2, 4, 24, 48 hours. (C and D) Primary microglia (n = 3) were treated with LPS (0, 1, 10, 100, 1000ng/mL) for 24 hours. The mRNA and protein levels of sPLA2-IIA were determined by western blot as described in methods. (TIF) [file pone.0077909.s001.tif]
